# L’insuffisance surrénalienne chronique: une cause sous-estimée de fatigue chronique

**DOI:** 10.11604/pamj.2018.29.93.14583

**Published:** 2018-01-30

**Authors:** Marie-Pierrette Ntyonga-Pono

**Affiliations:** 1 Faculté de Médecine de Libreville, Libreville, Gabon; 2 Service d’Endocrinologie au Centre Hospitalier Universitaire de Libreville, Gabon

**Keywords:** Fatigue chronique, insuffisance surrénalienne, corticoïdes, Chronic fatigue, adrenal insufficiency, corticosteroids

## Abstract

L'insuffisance surrénalienne chronique est considérée plutôt comme une maladie rare dans sa forme primitive. Par contre la forme secondaire, essentiellement à l'arrêt de l'usage des corticoïdes serait plus fréquente allant de 4,2% à 60% selon la pathologie traitée, mais souvent méconnue. Nous rapportons le cas d'une patiente de 66 ans qui se plaignait d'une fatigue chronique depuis plus de deux ans, allant de médecin en médecin et considérée à tort comme dépressive, alors qu'elle présentait en fait une insuffisance surrénalienne corticotrope.

## Abstract (en)

Chronic adrenal insufficiency is regarded as a rare disease in its primary stage. However, secondary adrenal insufficiency, which essentially occurs after stopping corticosteroids, is more frequent, ranging from 4.2% to 60%, depending on the pathology treated, but it is often unrecognized. We report the case of a 66 year old female patient with a history of chronic fatigue for more than 2 years. The patient consulted several clinicians but symptoms were wrongly attributed to a depressive disorder rather than to adrenal corticotropic adrenal insufficiency.

## Introduction

La fatigue est un symptôme banal puisqu'elle toucherait 1/3 de la population générale dans une étude néerlandaise [1] et sa prévalence augmenterait avec l'âge touchant plus de 50% de la population après 70 ans [2]. Bien qu'il n'y ait pas de définition consensuelle, on considère la fatigue comme un état extrême et persistant d'épuisement, de faiblesse, mentale et / ou physique [3]. Selon la durée, on distingue les épisodes de fatigue de courte durée de moins de 6 mois, la fatigue chronique depuis plus de 6 mois [1] qui est à distinguer du syndrome de fatigue chronique, entité bien précise [4]. Les causes les plus souvent évoquées peuvent être psychologiques, psychiatriques ou organiques allant des affections néoplasiques aux maladies auto-immunes, affections digestives, cardio-vasculaires neurologiques, endocrino-métaboliques dont le diabète sucré et les pathologies de la thyroïde [4]. Mais l'insuffisance surrénalienne est considérée comme une maladie rare dans sa forme primitive, avec une prévalence de 93 à 144 cas par million d'habitants en Europe [5]. Par contre l'insuffisance surrénalienne d'origine centrale dite corticotrope, serait plus fréquente, environ le double, en rapport le plus souvent avec l'arrêt d'un traitement prolongé par les corticoïdes [5]. Nous rapportons le cas d'une patiente de 66 ans qui a développé une insuffisance surrénalienne centrale, corticotrope, après infiltrations de corticoïdes, pour attirer l'attention sur cette pathologie sous-estimée en général [6] et qui peut décompenser sur un mode aigu et entrainer une issue fatale [5,7].

## Patient et observation

Mme O, âgée de 66 ans nous a consulté pour la première fois en Mai 2016, pour une fatigue chronique qu'elle trainait depuis deux ans environ, et pour laquelle elle avait vu plusieurs médecins qui lui disaient qu'elle n'avait rien d'anormal et la considéraient comme dépressive. Dans ses antécédents , on note une multiparité, six accouchements qui se sont tous bien passés, une hypertension artérielle bien contrôlée par Amlodipine 5mg par jour, des problèmes digestifs notamment des polypes du colon traités par voie endoscopique en France et un volumineux kyste biliaire drainé chirurgicalement en France où cette patiente se rend de temps en temps auprès de ses enfants qui y vivent. Elle rapporte aussi un épisode d'hyperglycémie transitoire traitée par Metformine pendant environ 6 mois. Elle avait été déclarée diabétique mais l'intolérance à la Metformine lui avait fait abandonner le traitement. Depuis lors elle faisait de temps en temps des contrôles de sa glycémie au laboratoire, qui restait normale

A l'examen clinique, elle était anxieuse, mais en assez bon état général. Sa plainte principale était cette fatigue qui ne la quittait pas et en lui demandant si c'était toujours pareil elle a répondu qu'elle se sentait un peu mieux le matin. Elle se plaignait aussi de douleurs erratiques, surtout abdominales, qu'elle rattachait à son kyste biliaire hépatique. Il n'y avait pas de mélanodermie. Elle pesait 58,5 kilogrammes pour 1,6om mais signalait un amaigrissement non chiffré. La pression artérielle était à120/80 mm Hg et le pouls régulier à 84/minute. A l'examen somatique systématique, nous avons relevé la présence de cicatrices chéloïdiennes au niveau de la poitrine au-dessus du sein droit un peu dépigmentées et aplaties. En la questionnant sur ces chéloïdes elle nous a expliqué que pendant des années elle a reçu des infiltrations locales de Kenacort *(triamcinolone) faites par une dermatologue, une fois par mois pour les faire disparaître. Elle avait arrêté parce que cela lui coûtait cher et ne semblait pas très efficace. L'examen cutanéo-phanérien, outre ces chéloïdes était normal. Il n'y avait pas de tâches pigmentées à la face interne des joues. La palpation abdominale retrouvait une masse épigastrique sensible, rénitente et le reste de l'examen systématique était normal. Sur le plan para clinique, le volumineux dossier qu'elle a apporté ne montrait rien de particulier. Les dernières glycémies étaient normales et nous avons demandé un dosage de l'hémoglobine glyquée (HbA1c) = 5,2%. L'hémogramme était normal, pas d'anémie pouvant expliquer cette fatigue, la sérologie rétrovirale était négative le bilan hépatique et rénal était normal ainsi que le ionogramme sanguin était normal. Le seul bilan hormonal qui avait été fait était le bilan thyroïdien (FT4 et TSH) qui était normal.

Nous avons complété ces explorations en demandant dans un premier temps un dosage du cortisol plasmatique à 8h qui est revenu bas 37 μg/l (microgrammes par litre) pour une normale entre 55 et 280 μg/l. Dans un second temps, un prélèvement pour dosage de l'ACTH a été envoyé en France (laboratoire CERBA) montrant une diminution à 3,0 ng/l (nannogrammes par litre) pour une normale entre 10,3 et 48,3ng/l). La patiente avait été mise sous hydrocortisone 30 mg/jour après le premier résultat et nous avons continué l'exploration hypothalamo -hypophysaire par la biologie et l'imagerie: prolactine normale, FSH LH valeurs de ménopause. Le scanner hypothalamo-hypophysaire avec injection a montré « une glande hypophysaire globalement homogène mais avec une légère asymétrie de volume au dépend du côté droit, ne permettant pas d'écarter un micro adénome hypophysaire». Une IRM était nécessaire, elle a été faite en France où cette patiente s'est rendue par la suite, mais elle était normale. Par ailleurs, l'échographie abdominale a montré de multiples kystes hépatiques dont le plus gros mesurait 10,4cm x 8cm, les surrénales n'étaient pas vues. Le diagnostic retenu a été celui d'insuffisance surrénale corticotrope secondaire probablement aux infiltrations prolongées de corticoïdes et qui a été confirmé par nos confrères en France. Depuis qu'elle est sous hydrocortisone la patiente a retrouvé son entrain et va bien.

## Discussion

Cette patiente présentait donc une insuffisance surrénalienne centrale, corticotrope par mise au repos de l'axe hypothalamo-hypophyso- surrénalien suite aux infiltrations répétées de triamcinolone, cependant il convient de souligner que toute corticothérapie prolongée quelle que soit la voie d'administration [8] est susceptible d'entraîner lors de son arrêt cette complication avec cependant des variations de prévalence selon les pathologies [6]. En effet chez les asthmatiques utilisant des corticoïdes par voie nasale, la prévalence de l'insuffisance surrénalienne centrale est de 4,2% mais atteint 50% lors des infiltrations articulaires et 60% lors du traitement des hémopathies malignes par des protocoles utilisant la corticothérapie [6]. Vu la grande utilisation de ces médicaments dans différentes spécialités médicales qui concernerait 1% de la population [6] nous croyons que la prévalence réelle de cette complication est sous-estimée. Il convient de sensibiliser les médecins pour qu'ils éduquent leurs patients et observent les règles d'arrêt d'une corticothérapie [5,7]. Dans les pays développés cela se fait probablement, mais dans nos pays en voie de développement, les corticoïdes sont utilisés de façon plus ou moins anarchique même par des paramédicaux qui font des infiltrations pour soulager les douleurs articulaires. Nous avons reçu plusieurs patients dans notre service d'endocrinologie pour des diabètes secondaires à ces infiltrations de corticoïdes, ce qui fut probablement aussi le cas de cette patiente que je n'ai pas vu au début de son histoire. Étant sensibilisés au problème, nous demandons systématiquement pour ces patients un dosage du cortisol plasmatique de base qui revient souvent abaissé. Nous ne faisons pas systématiquement de tests au synacthène [5] car le service est sous équipé et les dosages hormonaux se font dans des laboratoires privés au frais des patients qui n'ont pas toujours le moyens de les payer. Une exploration hypothalamo-hypophysaire hormonale et par l'imagerie est toujours nécessaire pour éliminer les autres causes d'hypopituitarisme notamment le syndrome de Sheehan, fréquent dans nos pays [9] et les tumeurs hypophysaires. La plainte initiale de cette patiente était une fatigue chronique, symptôme qu'il ne faut pas banaliser mais dont il faut préciser les caractères sémiologiques: type, intensité, horaire et variations dans la journée, facteurs qui l'influencent, autres signes associés éventuellement avant de conclure rapidement à une dépression. En effet la fatigue en cas de dépression, persiste au long de la journée et elle est associée à d'autres signes comme la tristesse, le repli sur soi, les troubles du sommeil, la perte de l'appétit, les idées suicidaires [4,10]. Pour aider à évaluer et classer une fatigue dans un cadre psychologique ou organique, diverses échelles plus ou moins complexes ont été mises au point [3,4], mais la séparation n'est pas toujours facile car la dépression peut accompagner plusieurs pathologies organiques [2].

## Conclusion

La fatigue chronique qui dure plus de 6 mois est un symptôme fréquent pouvant relever de causes organiques, psychologiques ou mixtes. Nous avons présenté le cas d'une patiente se plaignant d'une fatigue chronique et considérée comme dépressive alors qu'elle présentait une insuffisance surrénalienne centrale, secondaire à une corticothérapie prolongée. Vu la grande utilisation des corticoïdes en médecine, nous croyons que cette insuffisance surrénalienne chronique centrale est sous-estimée et nécessiterait une meilleure sensibilisation du personnel de santé.

## Conflits d’intérêts

L'auteur ne déclare aucun conflit d'intérêts.
